# Retrospective review of dosing trends in botulinum toxin injections for the treatment of adductor spasmodic dysphonia in a long-term cohort

**DOI:** 10.1186/s40463-020-0401-4

**Published:** 2020-01-14

**Authors:** Gabrielle French, J. Douglas Bosch, Derrick R. Randall

**Affiliations:** Section of Otolaryngology – Head & Neck Surgery, Department of Surgery, Cumming School of Medicine, University of Calgary, Calgary, AB T2W 3K2 Canada

**Keywords:** Spasmodic dysphonia, Botulinum toxin, Dysphonia

## Abstract

**Background:**

Botulinum toxin A (BT) is the gold standard treatment for adductor spasmodic dysphonia (AdSD) with established use for greater than thirty years. The spasmodic dysphonia (SD) literature would benefit from additional long-term cohort data, especially in the Canadian population. The goals of this study were to evaluate whether BT dosage required to achieve acceptable voice shifts over time and to elucidate differences in the subgroups of patients receiving unilateral vocal fold (UVF) injections.

**Methods:**

Patient records were retrospectively reviewed at the regional tertiary Voice Clinic for AdSD patients from 1996 to 2017 to identify AdSD patients treated with serial BT injections. Descriptive statistics, paired t-tests for time between treatments and ANOVA tests were used to evaluate trends in subgroup age.

**Results:**

One-hundred and twenty-six patients (61% female, mean age = 53 ± 15.5 years) met inclusion criteria and received laryngeal EMG-guided BT injections for up to twenty-two years and as many as 79 treatments. The mean total BT dosage for our population was 1.54 ± 0.35 Units per side. The majority of subjects had decreasing doses over time with a small subgroup having slowly increasing doses. Comparing treatment dosages between unilateral and bilateral injection groups, injection dosage per vocal fold was 1.65 ± 0.62 with time between injections was significantly shorter for the unilateral injection group (mean = 105 days, SD ± 19.8 days, *p* = 0.005) compared to the bilateral injection subgroup (137 ± 35.7 days, *p* < 0.005). The mean age of the unilateral injection population as younger at 42.4 ± 11.8 years (*p* = 0.004).

**Conclusion:**

The majority of patients in this study had decreasing BT injection dosages over time, with a smaller proportion having slowly increasing doses, thought to be likely relating to disease severity. The unilateral vocal fold injections were well tolerated despite needing more frequent injections, and found to be more prevalent in the younger age group.

## Background

Spasmodic dysphonia is a focal dystonia of the laryngeal musculature causing characteristic involuntary movements of the vocal folds. Although its etiology is not known, spasmodic dysphonia (SD) is thought to be a central motor processing disorder of the basal ganglia and its connections, which results in action-induced involuntary spams of the laryngeal musculature [1]. Spasmodic dysphonia has been found to have drastic effects on patient’s quality of life, particularly affecting them socially, functionally and emotionally [2]. With this profound impact effective treatment has made a substantial improvement in patient’s quality of life [3].

The gold standard treatment of this condition is botulinum toxin A (BT) injection into the laryngeal muscles, first described by Blitzer et al. [4–6] Since its discovery BT has been used in SD in a variety of doses [7]. The benefit of BT injections must be weighed against the temporary side effects of breathiness, weak voice, dysphagia, and weak cough. Unilateral vocal fold injections have been found to be effective to reduce side effects associated with BT injections in small populations [8, 9]. The literature on SD would benefit from addition of long-term cohort data, especially in the Canadian population [10–12]. There is also a question regarding the presence of acquired resistance to BT in the SD population stemming from the cervical dystonia and cosmetic literature [13–17]. As the regional tertiary voice centre providing treatment for Adductor Spasmodic Dysphonia (AdSD) in the health region we have an opportunity to identify patient specific factors related to BT injections over a long term Canadian cohort. With this study we sought to examine our own dosing patterns for BT dosing in individuals with AdSD in our population with a further subgroup analysis of those undergoing unilateral vocal fold injections.

## Methods

The University of Calgary Cumming School of Medicine Institutional Review Board provided ethical approval for this study (CHEB #REB16–2507). Patients treated for AdSD between 1994 and 2017 at the tertiary regional Calgary Voice Clinic in southern Alberta were identified. All patients were evaluated in our multidisciplinary clinic by a laryngologist and a speech language pathologist. Inclusion criteria included all males and females with AdSD who were eighteen years of age or older. Exclusion criteria included patients with abductor or mixed spasmodic dysphonia, or those who declined BT treatment. The diagnosis of spasmodic dysphonia was made by clinical evaluation and flexible videostroboscopy. At our centre treatment employed onabotulinum toxin A (Allergan Inc., Markham, ON) reconstituted with saline to a concentration of 20 Units/mL. Electromyography guidance was used to inject the thyroarytenoid-lateral cricoarytenoid muscle complex. At each subsequent follow up the patients were asked their perceived voice quality and side effects. Subsequently a decision regarding dose alteration was made. The primary outcome of this study was the BT dosage trends over time. The secondary outcome was a subgroup analysis of patients receiving UVF and BVF injections. Statistical analysis was performed with SPSS software version 25.0 (SPSS- IBM Corporation, Armonk, NY) using descriptive statistics, paired t-tests for time between treatments and ANOVA test for ages of subgroups.

## Results

In our retrospective chart review, 126 subjects (61% female) representing 3007 total injections met inclusion criteria. The average age at first treatment for all subjects was 52.7 years. Ninety-eight underwent bilateral vocal fold (BVF) injections for their first treatment, with mean dosage 1.3 ± 0.56 Units per side. Six percent of patients elected not to proceed with a second BT treatment and were lost to follow up. The median time in treatment was 6 years (range = 1–22 years). The median number of treatment sessions was 14 (range = 1–79). The average BT dose per vocal fold was 1.54 ± 0.35 Units. Time to reach a stable injection dose was found to be five injection appointments (1.8 years) in our cohort.

When considering UVF and BVF injection groups separately, 101 continued with BVF injections while 17 switched to UVF injections at some point during treatment. The average age at first treatment of the bilateral injection group was 54.4 ± 15.4 years, with the age at time of switch to UVF injections being significantly younger at 45.5 ± 13.5 years (*p* = 0.04). On average, patients who switched to unilateral injections changed on their 9th injection (range = 1–21 treatments). Ninety-four percent of the subjects who underwent UVF injections never switched back to BVF injections. The mean BT dose per vocal fold in the bilateral injection subgroup was 1.28 ± 0.41 Units, while the mean BT dose within the UVF injection group was 1.65 ± 0.62 Units. Of the BVF injection subjects who underwent more than ten injection sessions (*n* = 44), 74.6% had their dose decrease or stay the same from their optimized dose to their final dose. Those within the UVF group who received at least ten injections (*n* = 9) had a similar proportion, 75%, of those whose dose was stable or decreasing. Within both the UVF and BVF groups 25% of the subjects had an increasing dose from their optimized dose to their final dose. The mean increase in dose in the UVF group was 0.78 ± 0.41 Units. Within the bilateral vocal fold group the mean increase in dose was 0.54 ± 0.36 Units. There were no subjects in either group with continued exponential increases in their dose, suggesting acquired resistance did not play a role in our population.

The number of days between treatment visits were found to be shorter in the UVF injection group than BVF group with 105 ± 20 days and 139 ± 36 days respectively (*p* = 0.005).

## Discussion

Our study reviewed all available AdSD patients in our tertiary voice centre for BT dosing trends. With this we contribute to the literature by helping to identify BT dosages over time and how they may change. t also helps elucidate those who might particularly benefit from UVF dosing and which doses may work for them. We were able to capture long-term data from a tertiary voice clinic with a mean of eight years in treatment for our population. In terms of demographics, our population was in keeping with the literature with a slightly predominant female population of 61%. The average dose in our population per vocal fold of 1.54 ± 0.35 Units is in keeping with the literature [6]. The doses were mostly stable or decreasing over time which is also in keeping with previous long-term data [7, 15]. The smaller proportion of subjects whose doses increased, did so at a slow rate, indicating it was unlikely secondary non-response and more likely associated with disease severity, which is in keeping with data in the cervical dystonia population [16–19].

With regards to unilateral vocal fold injections, we found this cohort to be significantly younger at a mean age of 42.4 ± 11.8 years (*p* = 0.004), while the bilateral vocal fold injection group had a mean age of 54.4 ± 15.4 years with no significant difference in difference in gender. This study did not specifically assess voice outcomes due to the retrospective design; furthermore the voice outcomes and side effects were not explicitly recorded or measured by standardized patient reported outcome measures. However the dosing decisions were based on factors including voice outcomes, tolerance of side effects, laryngeal anatomy and operator experience. Our findings suggests that practitioners should keep unilateral vocal fold injections as an option, especially for younger patients. Further investigation into specific motivations for patients to select UVF injections and the patient-perceived impacts of improved quality of voice versus tolerability of side effects would be contributory to the SD literature.

Some of the limitations of this study include its retrospective nature which is associated with inherent biases and the aforementioned difficulty distinguishing dosing decisions as a result of voice benefit versus side effect dissatisfaction. Associated with this retrospective nature is the fact that a single otolaryngologist performed and counselled these patients and may have caused some bias to the subgroup analysis in possibly offering UVF injections to younger patients.

## Conclusion

The results of our study help contribute to the growing body of literature in long-term outcomes of BT injections in the AdSD population. We have been able to contribute to the dosing trends over time and have helped delineate trends within unilateral and bilateral vocal fold injection groups. This study continues to support further prospective research into the treatment of SD when looking at which individuals may benefit the most from unilateral or bilateral vocal fold injections.

## Data Availability

The datasets analyzed during the current study are available from the corresponding author on reasonable request.

## Abbreviations

AdSD: Adductor spasmodic dysphonia
BT: Botulinum toxin
BVF: Bilateral vocal fold
SD: Spasmodic dysphonia
UVF: Unilateral vocal fold

## References

[1] Hintze JM, Ludlow CL, Bansberg SF, Adler CH, Lott DG (2017). Spasmodic dysphonia: a review. Part 2: characterization of pathophysiology. Otolaryngol Head Neck Surg.

[2] Rubin AD, Wodchis WP, Spak C, Kileny PR, Hogikyan ND (2004). Longitudinal effects of Botox injections on voice-related quality of life (V-RQOL) for patients with Adductory spasmodic dysphonia: part II. Arch Otolaryngol Head Neck Surg.

[3] Gama ACC, Menezes LN, Maia AA, Rezende Neto AL, Oliveira JB (2010). Voice related quality of life after botulinum toxin injection for spasmodic dysphonia. Rev Laryngol Otol Rhinol (Bord).

[4] Blitzer A, Brin MF, Fahn S, Lovelace RE (1988). Localized injections of botulinum toxin for the treatment of focal laryngeal dystonia (spastic dysphonia). Laryngoscope..

[5] Watts CR, Truong DD, Nye C (2008). Evidence for the effectiveness of botulinum toxin for spasmodic dysphonia from high-quality research designs. J Neural Transm (Vienna).

[6] Watts C, Nye C, Whurr R (2006). Botulinum toxin for treating spasmodic dysphonia (laryngeal dystonia): a systematic Cochrane review. Clin Rehabil.

[7] Bradley JP, Barrow EM, Hapner ER, Klein AM, Johns MM (2017). Botulinum toxin-a dosing trends for adductor spasmodic dysphonia at a single institution over 10 years. J Voice.

[8] Bielamowicz S, Ludlow CL (2000). Effects of Botulinum toxin on pathophysiology in spasmodic dysphonia. Ann Otol Rhinol Laryngol.

[9] Bielamowicz S, Stager SV, Badillo A, Godlewski A (2002). Unilateral versus bilateral injections of botulinum toxin in patients with adductor spasmodic dysphonia. J Voice.

[10] Tisch SHD, Brake HM, Law M, Cole IE, Darveniza P (2003). Spasmodic dysphonia: clinical features and effects of botulinum toxin therapy in 169 patients-an Australian experience. J Clin Neurosci.

[11] Tang CG, Novakovic D, Mor N, Blitzer A (2016). Onabotulinum toxin a dosage trends over time for adductor spasmodic dysphonia: a 15-year experience. Laryngoscope..

[12] Blitzer A (2010). Spasmodic dysphonia and botulinum toxin: experience from the largest treatment series. Eur J Neurol.

[13] Berman B, Seeberger L, Kumar R (2005). Long-term safety, efficacy, dosing, and development of resistance with botulinum toxin type B in cervical dystonia. Mov Disord.

[14] Stephan F, Habre M, Tomb R (2014). Clinical resistance to three types of botulinum toxin type a in aesthetic medicine. J Cosmet Dermatol.

[15] Rosow DE, Pechman A, Saint-Victor S, Lo K, Lundy DS, Casiano RR (2015). Factors influencing botulinum toxin dose instability in spasmodic dysphonia patients. J Voice.

[16] Greene P, Fahn S, Diamond B (1994). Development of resistance to botulinum toxin type a in patients with torticollis. Mov Disord.

[17] Ferreira JJ, Colosimo C, Bhidayasiri R, Marti MJ, Maisonobe P, Om S (2015). Factors influencing secondary non-response to botulinum toxin type a injections in cervical dystonia. Parkinsonism Relat Disord.

[18] Smith ME, Ford CN (2000). Resistance to botulinum toxin injections for spasmodic dysphonia. Arch Otolaryngol Head Neck Surg..

[19] Park J-B, Simpson LL, Anderson TD, Sataloff R (2003). Immunologic characterization of spasmodic dysphonia patients who develop resistance to botulinum toxin. J Voice.

